# Patient and Payer Preferences for Additional Value Criteria

**DOI:** 10.3389/fphar.2021.690021

**Published:** 2021-06-24

**Authors:** Ivett Jakab, Melanie D. Whittington, Elizabeth Franklin, Susan Raiola, Jonathan D. Campbell, Zoltán Kaló, R. Brett McQueen

**Affiliations:** ^1^ Syreon Research Institute, Budapest, Hungary; ^2^ Department of Clinical Pharmacy, University of Colorado Anschutz Medical Campus, Aurora, CO, United States; ^3^ Cancer Support Community, Washington, DC, United States; ^4^ Real Endpoints, LLC, Florham Park, NJ, United States; ^5^ Center for Health Technology Assessment, Semmelweis University, Budapest, Hungary

**Keywords:** Multiple Criteria Decision Analysis, health technology assessement, value, patient, payer

## Abstract

**Background:** Defining the value of healthcare is an elusive target, and depends heavily on the decision context and stakeholders involved. Cost-utility analysis and the quality-adjusted life year (QALY) have become the method and value definition of choice for traditional value judgements in coverage and pricing decisions. Other criteria that may influence value are often not measured and therefore omitted from value assessments, or are only used to qualitatively contextualize assessments. The objective of this study was to engage two key stakeholders; patients and payers to elicit and rank the importance of additional value criteria, potentially assessed in Multiple Criteria Decision Analysis (MCDA).

**Methods:** This study consisted of a focus group with cancer patients (*n* = 7), including follow-up questions through an electronic survey, and in-depth phone interviews with payers (*n* = 5).

**Results:** For payers, value equated either with criteria that provided tangible benefits (from their perspective) such as new treatment options that respond to serious unmet need. For patients, population-level value equated to options that would potentially benefit them in the future and the value of hope. However, these criteria were seen by payers as difficult to measure and incorporate into objective decision making.

**Limitations:** The findings from this study are primarily limited due to generalizability. Due to the small sample size, it was outside the scope of this study to calculate a weight for each criterion that could be used as part of a quantitative MCDA.

**Conclusion:** MCDA, with particular attention to qualitative aspects, is an avenue to incorporate these additional criteria into value assessments, as well as provide an opportunity for reflecting the patient’s preferences in assessing the value of a treatment.

## Introduction

Defining the value of healthcare is an elusive target, and depends heavily on the decision context and stakeholders involved. Patient-level decision making ideally relies on the providers’ medical experience and the patient’s and their caregiver’s (i.e., close family member’s) individual preferences. When making healthcare decisions on the population-level a more complex set of values should be considered, by aggregating perspectives of various stakeholders including patients, caregivers, health care providers, payers (e.g., policy makers and insurance companies) and the broader society (Garrison et al., 2018).

Cost-utility analysis and the quality-adjusted life year (QALY) have become the method and value definition of choice for many value assessment frameworks for coverage and pricing decisions on the population-level (Reed et al., 2019; Westrich, 2019). Other criteria that may influence value for patients, their caregivers; even payers are often not measured and therefore omitted from value assessments, or are only used to qualitatively contextualize assessments. While experts in the field have encouraged inclusion of additional elements of value in future value assessment applications (Lakdawalla et al., 2018; Reed et al., 2019), there is a need to engage patients and other stakeholders to identify the importance of these value criteria that extend beyond QALYs or unweighted life-years (Perfetto et al., 2017). Two stakeholders are heavily involved with the decision-making process and its implications, yet may be presumed to have conflicting views on value–patients and payers. Eliciting and identifying the importance of additional value criteria (also referred to as “novel”) from the perspective of these stakeholders may further justify and promote evidence generation on value criteria not necessarily considered in traditional value assessment applications for use in emerging methods such as Multi-Criteria Decision Analysis (MCDA).

MCDA has been gaining popularity as a method for healthcare value assessment in the past decade, with long-standing applications in non-healthcare sectors (McQueen et al., 2019; Antioch et al., 2017; Marsh et al., 2014). MCDA can account for the multiplicity of criteria by analyzing and aggregating the performance of healthcare interventions on multiple criteria of influence (Phelps et al., 2018). A critical step of conducting an MCDA, and one that drives all subsequent steps and outcomes from the analysis, is criteria selection (Angelis and Kanavos, 2017). Criteria selection includes identifying and assembling criteria that define value. These criteria are later scored and deliberated as part of a qualitative MCDA (Baltussen et al., 2019). The criteria could subsequently be weighted and aggregated in a quantitative MCDA (Baltussen et al., 2019).

The transparency and objectivity of population-level decisions can be supported by MCDAs designed for repeated use with a standard criteria set (Neumann et al., 2018). Whereas MCDA can also be implemented in the patient-level decision-making process between patients and providers. One-off MCDA can be used as a tool to highlight potentially valuable criteria, facilitate ongoing treatment decision discussions (as the concepts a patient values may change with time), and ultimately both inform and empower patients and guide providers as they seek to provide patient-centered care (Neumann et al., 2018).

The objective of this study was to engage two key stakeholders–patients who incur the majority of health benefits from a therapeutic intervention and payers who make the majority of reimbursement decisions—to elicit and rank the importance of additional value criteria, not traditionally included in value assessment, that can inform future qualitative or quantitative MCDA applications to support population-level decisions. This work encompassed three activities, including: 1) capturing core elements of value from the oncology patient perspective in patient-level decision-making; 2) objectively assessing additional value criteria for population-level decision making, from a payer and patient perspective; and 3) comparing and contrasting findings between both stakeholders. Each activity is further defined in the methods section.

## Methods

This qualitative, multi-phase study was conducted as part of the pValue initiative, a research initiative at the University of Colorado Anschutz Medical Campus dedicated to apply and test novel methods for value assessment that encourages stakeholder engagement and promotes value-based decision making. The pValue initiative is financially supported by The PhRMA Foundation and the University of Colorado Data Science to Patient Value Initiative who reviewed the grant submission and objectives. PhRMA provided supplemental funding for logistical aspects, specifically, funding for structured interview planning and participation.

**FIGURE 1:**
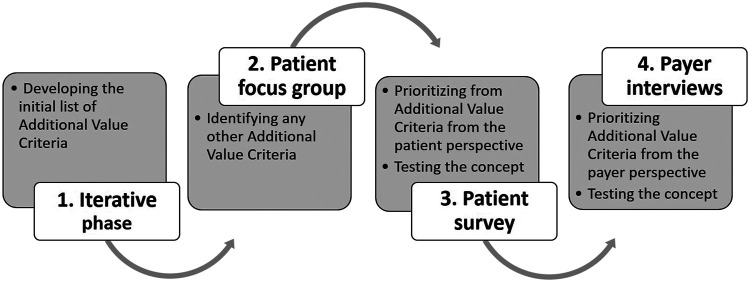
Structure of the research phases and aims.

The study consisted of 1) a preparatory iterative phase, 2) a focus group with cancer patients including 3) follow-up questions through an electronic survey, and 4) in-depth phone interviews with payers. The detailed structure and aims of each phase can be found on Figure 1.

The study was approved by the Colorado Multiple Institutional Review Board under number #19-0779 (Melanie Whittington as contact).

### Iterative Phase

**FIGURE 2:**
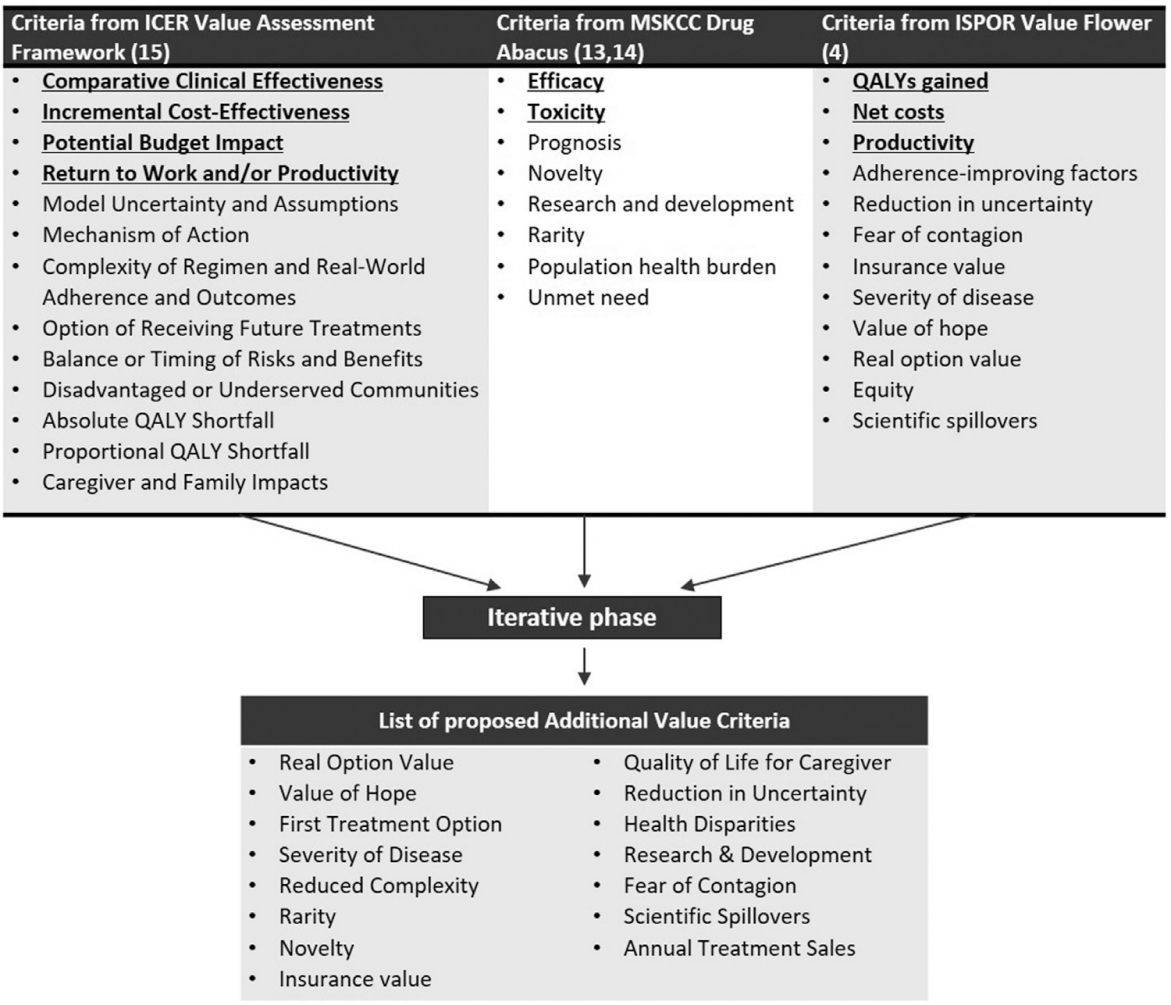
Extracted value criteria from United States value frameworks and the list of proposed Additional Value Criteria after the iterative phase. Institute for Clinical and Economic Review (ICER); International Society for Pharmacoeconomics and Outcomes Research (ISPOR) now known as The Professional Society for Health Economics and Outcomes Research; Memorial Sloan Kettering Cancer Center (MSKCC). *Value criteria deem by the research team as traditionally (not necessarily systematically) assessed are bolded and underlined.

In the preparatory phase of the study the initial list of “additional value criteria” was developed to be ranked and tested on patients and payers. Criteria were aggregated from the Memorial Sloan Kettering Cancer Center Drug Abacus (Djatche et al., 2018; Lanitis et al., 2019), the ISPOR value flower (Lakdawalla et al., 2018), and the Institute for Clinical and Economic Review’s Other Benefits and Contextual Considerations (Institute for Clinical and Economic Review (ICER), 2020). These three frameworks were selected from the findings of a systematic literature review of value frameworks (Jakab et al., 2020). Those frameworks published from the United States context including any additional value criteria were chosen. Categorization of the criteria as traditional or additional was based upon the judgement pValue research team through an iterative process. The aggregated criteria from the three frameworks, their categorization and the developed list of initial additional value criteria can be found on Figure 2.

### Patient Focus Group Interview

In collaboration with researchers with professional social work backgrounds and qualitative methods expertise from the Cancer Support Community, the largest nonprofit provider of psychosocial oncology support services in the United States, a focus group guide was developed. Adult patients (aged ≥ 18 years) diagnosed with any type of cancer and recruited through Cancer Support Community. Inclusion criteria were basic by design as we did not want to restrict our sample. The purpose of the focus group interview was to engage patients on criteria that are commonly considered when conceptualizing the value of oncology therapies in general. Thereby, the primary question for the patient focus group was, “If you were diagnosed with an illness today, what factors would you consider when making your treatment decision?” Adult patients who had previously received a cancer diagnosis were recruited through an affiliate of the Cancer Support Community. The focus group was held in person and was moderated by a trained facilitator and followed the structured interview guide (Supplementary Material 1). Note that the interview guide was used as a general guide for the discussion, but deviations were allowed. The focus group was recorded and transcribed. The raw data were read in detail by two researchers. Inductive coding of the data occurred simultaneously by each researcher. Initial lists of codes were created, refined through focused coding, and ultimately compared for consistency. Thematic analyses took place in order to determine linkages between codes and inclusion of all salient information (Padgett, 2016). Researchers discussed and aligned on over 95% of codes and common themes and negative case analysis occurred.

### Electronic Follow-Up Survey for Patients

An electronic survey was emailed to each patient participant following the focus group (Supplementary Material 2). The intent of this electronic survey was to focus on criteria that are less commonly considered, but might be important to consider when thinking about the value of a treatment on the population level.

The description of the 15 value criteria presented for the participants can be found in Table 1. Participants were asked to rank order the five most important factors that payers (e.g., policy makers and insurance companies) should consider when thinking about the value of a treatment. Participating patients were prompted to approach the survey questions from a broader societal perspective rather than focusing on their personal experience. This limiting shift in perspectives was deem necessary because a number of the proposed criteria are difficult to interpret on the individual level (e.g., Health Disparities, Scientific Spillover). A rank-based algorithm was then used to aggregate and rank order the criteria across the participants. The criterion ranked #1 by a participant received five points; the criterion ranked #2 by a participant received four points; the criterion ranked #3 by a participant received three points; the criterion ranked #4 by a participant received two points; the criterion ranked #5 by a participant received one point. Based on these points, an aggregated score was calculated for each criterion which was transformed into the overall ranking of all patients participating. Aggregated scores of each criterion are not published in the paper, because weighting criteria was outside of the scope of this study.

**TABLE 1:** Additional Criteria with top five ranking by patients and payers.

Criteria	Definition	Patient ranking	Payer ranking
Real option value	Potential for a treatment to extend life and create opportunities to benefit from other future advances in medicine	1	−
Value of hope	Potential for a treatment to provide a chance at a “cure”	2	−
First treatment option	The treatment is the first to offer any improvement for patients with a certain disease	3−4	3
Severity of disease	The severity (e.g., impact on length of life and/or quality of life) of a disease the treatment is intended to treat	3−4	2
Reduced complexity	The potential for a treatment to be simpler (e.g., in administration, simpler dosing, etc.)	5	4
Rarity	Potential for a treatment to address a rare disease that only affects a small percentage of the population	−	1
Novelty	New treatment option for patients for whom other available treatments have failed	−	5−6
Insurance value	Potential for a treatment to provide protection from physical risks of illness and financial risks of treating disease	−	5−6
Quality of life for caregiver	The quality of the caregiver’s daily life, including all emotional, social, and physical aspects	−	−
Reduction in uncertainty	New evidence that could better predict treatment outcomes	−	−
Health disparities	Potential for a treatment to reduce important inequalities across racial, ethnic, gender, socioeconomic, or regional categories	−	−
Research and development	The costs required to research and develop a new treatment	−	−
Fear of contagion	Potential for a treatment to address the anxiety/fear associated with the spread of disease	−	−
Annual treatment sales	Expected total yearly sales of the treatment	−	−
Scientific spillovers	The potential impact a treatment could have on future research and development	−	−

### Payer in-Depth Interviews

Second, we conducted five (National Plans *n* = 3; Regional Plans *n* = 2), 45-min in-depth phone interviews with United States-based medical directors from national and regional health plans. The interviews were conducted between February 17 and March 5, 2020 following the guide in Supplementary Material 3. The primary goal of this research was to understand what payers consider when evaluating the value of a treatment, outside of traditional value criteria. Note that as opposed to patients, payers did not discuss oncology specifically. The same 15 value criteria were discussed in detail and ranked by payers following the same ranking procedure completed by the patients (Table 1) and aggregated with the same rank-based algorithm. Additionally, payers gave an insight in which value criteria they thought were routinely assessed by payers in the context of coverage and reimbursement decisions of pharmaceuticals, which criterion’s definitions should be refined, and which criteria should be removed from the list.

## Results

### Patient Results

The in-person focus group was held on October 18th, 2019 at the Cancer Support Community Delaware affiliate and lasted 105 min. Seven adult patients (Six women; one man) participated in the focus group. The participants had all previously received a cancer diagnosis, including breast (*n* = 3; 43%), non-Hodgkin lymphoma (*n* = 1; 14%), ovarian (*n* = 1; 14%), prostate (*n* = 1; 14%), and tongue cancer (*n* = 1; 14%). Throughout the discussion, participants indicated that survival was the most highly valued factor when choosing a treatment. One participant said, *“I’ll say that because I had young kids it was very important to me to be around for the kids as long as possible”.* Focus group participants also emphasized quality of life and the ability to provide for their family when making their treatment decisions. For quality of life, another focus group participant stated, *“Once it metastasized and there was no way of getting a cure, it came down to quality of life. Was I going to be miserably sick throwing up every other day?”* Focus group participants also emphasized productivity, with one participant stating, *“I still feel pressure to do what I can to take care of my wife and son for the future”.* Focus group participants also noted multiple provider-level attributes that influence their decision making, including access to providers, quality of care, and communication. Importantly, no following criteria were identified as Additional Value Criteria to feed the prioritization exercise in the follow-up survey.

Subsequent surveys were emailed to the respondents in December 2019. Survey responses were received from six of the participants (86%). Table 1 presents the rank order assigned to each additional value criteria from the patient survey efforts. The patients ranked the following criteria as the most important for payers to consider when assessing the value of a treatment: #1 Real Option Value, #2 Value of Hope, #3-4 First Treatment Option, #3-4 Severity of Disease, and #5 Reduced Complexity.

### Payer Results

Payers were asked to review the list of 15 additional value criteria and their corresponding definitions shown in Table 1. Additionally, they were then asked to rank the top five additional value criteria in order of importance when considering the value of drug/treatment. Payers ranked the following additional value criteria as most important ones: #1 Rarity; #2 Severity of Disease; #3 First Treatment Option; #4 Reduced Complexity; #5-6 Novelty; #5-6 Insurance Value (Table 1).

Most of the payers thought Rarity was the most important factor since it provides treatment and represents the current standard of care with reasonable outcomes when nothing is available for rare/orphan indications, also reflecting current evaluation of value. *“The first one would be Rarity. There has been a lot of attention recently about the treatment of orphan designated disease or ultra-orphan designated disease where there haven’t been any specific treatments currently in use and are not FDA approved but represents the standard of care that presently exists. A new drug comes in with FDA approval, the biggest problem is pharma uses orphan or ultra-orphan in the category to justify their cost. Six-figure price for a drug on a yearly basis often comes with some questionable outcomes.”* Severity of Disease was ranked second due to the reduction of disease symptoms in both severity and frequency.

Some said First Treatment Option was valuable because it may be the first treatment available for a particular disease and may represent the standard of care. Also, Reduced Complexity was regarded as routinely discussed, but not usually defined the way presented. According to them, Reduced Complexity was generally more about convenience (administration) and simpler treatments. *“Lots of drugs come [to the market] are once a day or sub Q instead of IV or rapid administration. So, we will always look at reducing complexity.”* Payers felt Insurance Value was important since it was closest to the cost of healthcare delivery. Finally, Novelty was interpreted similarly to Rarity of a drug. *“We focus on allowing benefits for the right treatment for the right patient at the time. And we want to deal [with the pharma companies] and provide the best care. So, if there’s nothing else out there like this novel is rare, we have to pay attention. We would rather pay for something that has novelty and rarity with reasonable outcomes.”*

Payers felt the majority of the additional value criteria definitions (Table 1) were appropriate. The following factors were flagged as needing additional clarity: Reduced Complexity, Scientific Spillover, and Value of Hope were not clearly defined. Payers felt that some of the additional value criteria should be categorized as traditional value criteria since they are routinely looked at when evaluating drugs/treatment: First Treatment Option/Novelty, Severity of Disease, Quality of Life of Caregivers (depending on the disease, i.e., Alzheimer), Rarity, and Reduced Complexity.

Payers recommended “Pediatrics” as an additional value criterion since many pediatric conditions have high morbidity. The payers named several of the additional value criteria that were unknown to them, and others that are difficult to measure the value of and should, therefore, be removed from the list: Fear of Contagion, Reduction in Uncertainty, Research and Development, Scientific Spillover, and Value of Hope.

In general, many of the payers said that these non-traditional criteria values did not significantly impact the way payers evaluate drugs. They believed that these criteria were more likely to resonate with pharmaceutical companies in terms of how they view the value of a drug, and do not necessarily relate to how payers assess it*.*

## Discussion

### Comparison of Patient and Payer Preferences

Despite different perspectives and decision contexts between patient and payers, significant overlap in preferences were revealed. Severity of Disease, First Treatment Option and Reduced Complexity made the priority list of both stakeholder groups, even though payers considered all three criteria as routinely assessed when evaluating treatments, thereby advising that these should to be recategorized as traditional criteria. The same comment was mentioned for two other criteria prioritized by payers as well, which were Rarity and Novelty, with the only exemption being Insurance Value. This finding is in line with the intention of the payer interviews; to identify the criteria currently considered, however, may not assessed systematically, in a measurable, objective manner. Our definition of traditional criteria in this study covered those elements of value which can be fully captured by the conventional cost-effectiveness approach, therefore the re-categorization on this basis seems unnecessary.

The top preference of patients was Real Option Value, defined as “Potential for a treatment to extend life and create opportunities to benefit from other future advances in medicine.” This domain did not make the payer’s list, but was not flagged either which suggests Real Option Value was clearly defined but not deem important by payers, as opposed to being a priority for patients. Value of Hope, the second highest scored value element for patients on the other hand was criticized heavily during payer interviews. Payers not only thought the definition “Potential for a treatment to provide a chance at a “cure”’ needed clarification, but some also suggested that Value of Hope should be removed from the list of potential value elements entirely. Their main concern was the lack of measurement for the criterion. According to an ICER Commentary for their Value Assessment Framework (Jakab et al., 2020), methods for empirically integrating value dimensions like Value of Hope into a value-based price are not well established and are viewed by many health economists as too exploratory for routine incorporation into assessments. On the other hand, ICER mentions that Value of Hope may be tied empirically to the risk attitudes of patient groups that vary widely depending on the severity of the condition and the prospects for future treatments to be effective.

Rarity was ranked as the most important value element for payers, but did not make it to the patients’ top five list.

Another value element ranked in the top five by payers, but not by patients was Insurance Value. Payers stated that Insurance Value was important since it is most closely related to the cost of healthcare delivery.

For payers, value equated either with criteria that provided tangible benefits (from their perspective) such as new treatment options that respond to unmet need, reduced complexity, or protection of patients from individual financial risk. For patients, value in their own treatment equated to criteria that were associated with their personal cancer journey or trajectory for improved survival, quality of life or productivity. When asked to consider population-level decision making patients valued options that would benefit them in the future and the value of hope. However, these criteria were seen by payers as difficult to measure and incorporate into objective decision making.

### Refinement of Criteria

Criteria and their definitions were used in this study as presented in the referenced value frameworks. However, as a potential next step, these criteria could be refined to reduce overlaps and/or clarify meaning. As an example, payers suggested that the criteria Novelty and First Treatment Option could be merged to avoid double counting. Another perspective provided by payers was to add Pediatrics as a new value criterion. Criterion Pediatrics and Rarity could even be considered to be merged into one single criterion, called “Vulnerable and/or Underserved Patient Populations”. This merging would not only help in avoiding double counting–many pediatric diseases are also rare–but to be more inclusive with other potentially neglected sub-populations like pregnant women, patients living with psychiatric illness, or patients living in rural areas.

### Implementation of Results

There are two main, non-mutually exclusive ways to increase the patient centricity of population-level decision making in healthcare. Patient representatives can either be involved in the value judgement directly, on a case by case basis, or value assessment frameworks guiding the decision-making process can be extended with patient-relevant criteria beyond the traditional value judgement. Ideally, the two methods are used in parallel and engaged patient representatives can provide qualitative and/or quantitative data on these patient-relevant value elements for each specific decision.

To make sure value frameworks are capturing what is valuable for patients, many societies and advocacy groups have called for additional engagement of patients in value assessment framework development (Diaby et al., 2019; Marc Boutin 2016). If implemented meaningfully, involving the patient perspective may result in better treatment experience/health outcomes more aligned with patients’ treatment goals, better use of resources and the potential for broader patient acceptance of payer decisions (Addario et al., 2018). The findings from this study highlight different ways in which patients and payers conceptualize value in health care. For patient-centric value assessment frameworks supporting population-level decisions, a resolution is needed to capture additional value elements important to patients, but deemed by payers to be difficult to currently measure. MCDA might aid this process in multiple ways.

There are no methodological guidelines to provide clear directions on how to ensure credibility and representativeness of the MCDA results by handling the stakeholders’ engagement in an appropriate manner (Kolasa et al., 2018). There are good practices in engaging large groups of payers and other healthcare decision-makers (Tanios et al., 2013). However, when engaging patients, generalizability is an issue not only in terms of the sample size and heterogeneity of preferences, but also in terms of the heterogeneity of diseases. Thoroughly capturing the “general patients” preferences’ or even “oncology patients” preferences’ is a considerable challenge as it would require recruiting patients from all or most different disease areas in a representative manner to the frequency of these diseases in the population. A way forward could be to define value not for the general patient population, but for patients with the same diagnosis to inform decision-making on new treatments for that specific patient sub-population. For future research, we aim to replicate this study with patients from multiple different disease groups in separate focus groups, and analyze the within- and between-group differences in their weighting of the same elements of value. Another challenge in patient engagement in MCDA development is framing the context of the question in terms of individual vs. population-level perspectives. Other stakeholders are requested to provide their value preferences practiced in their professional role (e.g., payer, health care practitioner), and even though their preferences will be inevitably influenced by their personal experiences outside their profession, the two are easier to distinguish. For patients, the stakeholder role they are asked to represent is closely connected to their personal experience with their health condition, which can introduce certain biases. For example, a non-rare disease patient might value “Rarity” low from their individual perspective, as it is not relevant for them. This challenge can be solved in two ways: patients can be asked to try to express their values from the societal perspective, or they can be asked to express their individual preferences only on those criteria, which might be relevant from an individual patient’s perspective. For this study, we chose the former solution, but we recommend researchers to experiment with both.

Almost two-thirds of value assessment frameworks published between January 2013 and March 2019 had no scoring functions and one-third of all frameworks did not have definitions, only the list of criteria names (Jakab et al., 2020). An MCDA tool designed for repeated use with clear definitions, rationale for inclusion, and well-defined scoring functions can provide a solution as we address the need for objective assessment and the challenges of available measurement methods in their infancy. Such MCDA tools can be used as an overall framework for all criteria; or as an extension of current decision making practices, only including criteria hard to capture otherwise (e.g., patient centric or societal value criteria). The calculation of an overall score is recommended, however, setting a decision rule is optional. MCDA can also be implemented into the decision-making process as a tool supporting the deliberate process, objectively examining the comprehensive value of a new treatment.

### Limitations

The findings from this study are primarily limited due to generalizability, but provide important insights as the science of MCDA progresses. The patient focus group was limited to patients who had previously received a cancer diagnosis, and the selection of patients was not representative of the variety or frequency of cancer diagnoses. Although appropriate for the research method, the study also had a relatively small sample size of patients. Therefore, ranking results of the patient survey should not be directly used to inform decision-making. In an effort to reduce heterogeneity between the participants, the external validity of these findings may be reduced. The patient focus group identified survival and quality of life, consistent with traditional criteria, as priorities in their judgement of a treatment’s value. The study design did not allow for comparisons of the relative importance of traditional vs. additional criteria from either the patient or payer perspective. Due to the small sample size, it was outside the scope of this study to calculate a weight for each criterion that could be used as part of a quantitative MCDA. However, this work is instrumental for reducing the number of criteria in order to conduct future weighting exercises (Németh et al., 2019). Future work will also engage larger groups of stakeholders across disease areas to refine criteria and elicit weights between and within each criterion. Also, the development of a rationale for inclusion and well-defined, criterion specific scoring function is recommended for all criteria to be included in a future MCDA.

### Conclusion

The emphasis on incorporating supplemental criteria beyond the QALY in population-level value assessment is intensifying. MCDA, with particular attention to qualitative aspects, is an avenue to incorporate these additional criteria into value assessments, as well as provide an opportunity for reflecting the patient’s preferences in assessing the value of a treatment. MCDA creation, implementation, and evaluation provide opportunities not only for improving the patient-centricity of formal value assessments but also an avenue for collaboration between health economists and patient advocates as they seek the achieve the parallel goal of efficient distribution of health resources in a way that is respectful to patient needs and transparent to all stakeholders.

## Data Availability

The original contributions presented in the study are included in the article/Supplementary Material, further inquiries can be directed to the corresponding author.

## Abbreviations

QALY, quality-adjusted life year; MCDA, Multi-Criteria Decision Analysis
