# Supplementary material for: Patient and Payer Preferences for Additional Value Criteria
Source: Front Pharmacol. 2021 Jun 24;12:690021. doi: 10.3389/fphar.2021.690021 (PMC8263917; doi:10.3389/fphar.2021.690021)
Supplement: Supplementary file 1 [file DataSheet1.docx]

*Supplementary Material 1. Patient Focus Group Interview Guide*

**INTRODUCE TOPIC OF RESEARCH**

We are holding this focus group to help us better understand the factors that are most important to patients when making healthcare decisions. We would really like to understand your experience as you were diagnosed with cancer and made treatment decisions. This discussion should last about an hour and a half.

As a bit of background, researchers from the University of Colorado and Cancer Support Community are partnering on a study to better understand treatment decision making for patients who have a current or past diagnosis of cancer. Ultimately, we want to understand what you “valued” at the time of treatment or care decisions and what you “value” today. By value, we are looking for the factors that are the most important for you and your wellbeing.

We’ll guide you through a discussion so that we can understand your experience with your health care.

**CONFIDENTIALITY AND GROUP NORMS**

As indicated in the informed consent form we just went through, I want to assure you that no one except the research investigators and the people here in this room with you will know that you took part in this discussion. We are recording the group discussions, but voices only, and the recordings will only be used to write down and consider the information you shared with us during the discussion. Your names will not be recorded in these notes. We ask that no one share any information they may have heard here with anyone outside the group.

Nothing that you say will be attributed to you or any other participant; this means none of your words will be reported as being said by you. Sometimes there will be a specific statement that we will share word-for-word in the final report or in other documents based on these discussions, including publications from the research; however, no names will be attached to the quote and it will remain anonymous. Your identity will not be revealed in any description, publication, or research. We will protect your personal information, and if the results of this research are published, no one’s name will be used. Does anyone have any questions about confidentiality?

For this discussion group, I will be asking several questions, and we encourage participation from everyone in answering and talking about the topics. There are no right or wrong comments or answers in this discussion, and all our experiences and perspectives are real and valued.

Please speak loudly and clearly and one at a time. Please turn your cell phone ringers off or to vibrate and put your phones away, since it is important for us all to be really present during these discussions.

Are there any questions before we get started?

**Introductions**

What kind of cancer(s) have you been diagnosed with and at what age(s)?

**Focus Group Questions**

1. Tell me about your diagnosis.
   1. What do you remember?
   2. What was that moment like?
   3. How did your parents/caregivers respond to the news?
   4. How involved were your parents/caregivers?
2. Did your caregivers/providers involve you in treatment decision making?
3. What changed in your life after your diagnosis?
4. When making treatment decisions, what is most important to you? In other words, what do you care about and “value?”
5. Were there times within your treatment or care where you had to make or be part of a difficult treatment or care decision? If so, please describe the decision and why it was difficult.
6. Knowing what you know today, would you have made any changes in your treatment or care?
7. If you were diagnosed with cancer or another illness today, what factors would you consider when making your treatment decision?
8. [provide handout] Please look over the sheet I just handed out. This sheet lists factors that may be valued in healthcare decision making.
   1. Are there other factors you would consider when thinking about the value of a treatment?
   2. Are there factors you would remove from this list?
   3. Out of this list, what is the most important to you? The least important to you?

**Focus Group Conclusion**

Thank you for all your time and for sharing your experiences with myself and one another so freely. This has been extremely helpful.

Please remember that you agreed to keep this discussion confidential. Please do not give details of what was said here, so that we can try to preserve the confidentiality of those who agreed to share their stories and experiences today.

Do you have any questions for me? If anyone would like to speak with me in private, I will stay here after we end.

Thanks again for all your help.

*Supplementary Material 2. Patient survey template*

Thank you for joining our focus group on October 18^th^ at the Cancer Support Community Delaware. We are thankful for the information you provided and look forward to sharing our findings with you. After the focus group, we had a few follow-up questions we were hoping to ask you. These questions are designed for you to help us understand what decision makers (such as policy makers and insurance companies) should consider when thinking about the value of a treatment. Instead of focusing on your personal experience and what matters to you personally, approach these questions more broadly and not specific to your diagnosis or treatment.

There are factors that are commonly considered when thinking about the “value” of a treatment. These include survival, quality of life, complications, and return to work. Below is a list of factors that are less commonly considered, but might be important to consider.

| **Factor** | **Definition** |
| --- | --- |
| Annual Treatment Sales | Expected total yearly sales of the treatment |
| Fear of Contagion | Potential for a treatment to address the anxiety/fear associated with the spread of disease |
| First Treatment Option | The treatment is the first to offer any improvement for patients with a certain disease |
| Health Disparities | Potential for a treatment to reduce important inequalities across racial, ethnic, gender, socioeconomic, or regional categories |
| Insurance Value | Potential for a treatment to provide protection from physical risks of illness and financial risks of treating disease |
| Novelty | New treatment option for patients for whom other available treatments have failed |
| Quality of Life for Caregiver | The quality of the caregiver’s daily life, including all emotional, social, and physical aspects |
| Rarity | Potential for a treatment to address a rare disease that only affects a small percentage of the population |
| Real Option Value | Potential for a treatment to extend life and create opportunities to benefit from other future advances in medicine |
| Reduced Complexity | The potential for a treatment to be simpler (e.g. in administration, simpler dosing, etc.) |
| Reduction in Uncertainty | New evidence that could better predict treatment outcomes |
| Research & Development | The costs required to research and develop a new treatment |
| Scientific Spillovers | The potential impact a treatment could have on future research and development |
| Severity of Disease | The severity (e.g. impact on length of life and/or quality of life) of a disease the treatment is intended to treat |
| Value of Hope | Potential for a treatment to provide a chance at a “cure” |

1. Of these factors, please rank order the five most important factors that decision makers should consider when thinking about the “value” of a treatment.
   1. 1-
   2. 2-
   3. 3-
   4. 4-
   5. 5-
2. Please explain your selections to question #1.
3. Are there any factors that should be removed from the list? If so, please list and explain.

*Supplementary Material 3. Payer interviews guide*

1. **Introduction**
   - Thank you for agreeing to participate in our market research study.
   - Confidentiality of the respondent: to maintain anonymity, please do not say your name or the name of your organization.
   - We are recording the call, for note taking purposes.
   - Questions are meant to facilitate discussion - no right or wrong answers to questions – need honest feedback
   - Our discussion today will focus on the value of treatment
   - These questions are designed for you to help us understand what decision makers (e.g. payers) should consider when thinking about the value of a treatment.

There are factors that are commonly considered when thinking about the “value” of a treatment. These include survival, quality of life, complications, and return to work. Below is a list of factors that are less commonly considered, but might be important to consider.  Let’s look at a list of factors that are less commonly considered when thinking about the value of a treatment but might be important to consider.


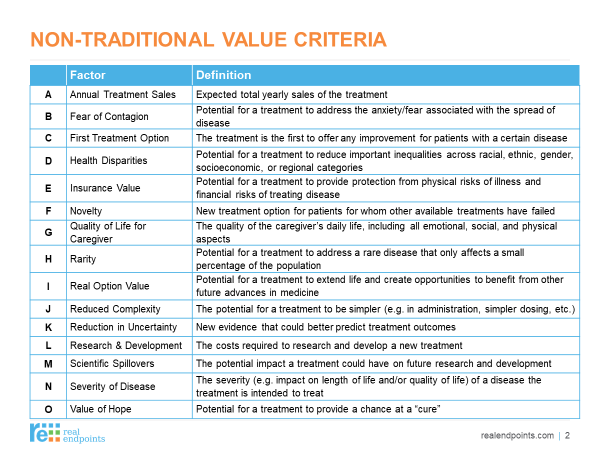


1. Are these the definitions you anticipated for each of these terms?
2. Are there any terms/definitions that are new for you?
3. Are any of the terms or definitions unclear or would you re-word any of them?
4. How do these factors compare to the more traditional factors we just discussed? Do all of them belong on this list or would you prioritize them differently (in other words, move any of them to the traditional factors list)?
